# Nuclear alpha-synuclein is present in the human brain and is modified in dementia with Lewy bodies

**DOI:** 10.1186/s40478-022-01403-x

**Published:** 2022-07-06

**Authors:** David J. Koss, Daniel Erskine, Andrew Porter, Pawel Palmoski, Hariharan Menon, Olivia G. J. Todd, Marta Leite, Johannes Attems, Tiago F. Outeiro

**Affiliations:** 1 Translational and Clinical Research Institute, Faculty of Medical Sciences, Newcastle University, Framlington Place, Newcastle Upon Tyne, NE2 4HH UK; 2 Newcastle University Protein and Proteome Analysis Unit, Newcastle University, Newcastle Upon Tyne, UK; 3 Department of Experimental Neurodegeneration, Center for Biostructural Imaging of Neurodegeneration, University Medical Center Goettingen, Göttingen, Germany; 4 Max Planck Institute for Multidisciplinary Sciences, Göttingen, Germany; 5 Scientific Employee with an Honorary Contract at German Center for Neurodegenerative Diseases (DZNE), 37075 Göttingen, Germany

**Keywords:** Alpha-synuclein, Dementia with Lewy bodies, Lewy body disease, Synucleinopathies, Nuclear pathology

## Abstract

Dementia with Lewy bodies (DLB) is pathologically defined by the cytoplasmic accumulation of alpha-synuclein (aSyn) within neurons in the brain. Predominately pre-synaptic, aSyn has been reported in various subcellular compartments in experimental models. Indeed, nuclear alpha-synuclein (aSyn^Nuc^) is evident in many models, the dysregulation of which is associated with altered DNA integrity, transcription and nuclear homeostasis. However, the presence of aSyn^Nuc^ in human brain cells remains controversial, yet the determination of human brain aSyn^Nuc^ and its pathological modification is essential for understanding synucleinopathies. Here, using a multi-disciplinary approach employing immunohistochemistry, immunoblot, and mass-spectrometry (MS), we confirm aSyn^Nuc^ in post-mortem brain tissue obtained from DLB and control cases. Highly dependent on antigen retrieval methods, in optimal conditions, intra-nuclear pan and phospho-S129 positive aSyn puncta were observed in cortical neurons and non-neuronal cells in fixed brain sections and in isolated nuclear preparations in all cases examined. Furthermore, an increase in nuclear phospho-S129 positive aSyn immunoreactivity was apparent in DLB cases compared to controls, in both neuronal and non-neuronal cell types. Our initial histological investigations identified that aSyn^Nuc^ is affected by epitope unmasking methods but present under optimal conditions, and this presence was confirmed by isolation of nuclei and a combined approach of immunoblotting and mass spectrometry, where aSyn^Nuc^ was approximately tenfold less abundant in the nucleus than cytoplasm. Notably, direct comparison of DLB cases to aged controls identified increased pS129 and higher molecular weight species in the nuclei of DLB cases, suggesting putative pathogenic modifications to aSyn^Nuc^ in DLB. In summary, using multiple approaches we provide several lines of evidence supporting the presence of aSyn^Nuc^ in autoptic human brain tissue and, notably, that it is subject to putative pathogenic modifications in DLB that may contribute to the disease phenotype.

**Supplementary Information:**

The online version contains supplementary material available at 10.1186/s40478-022-01403-x.

## Introduction

The hallmark pathology of Lewy body diseases (LBDs), including Parkinson’s disease (PD) and dementia with Lewy bodies (DLB), is the intraneuronal accumulation of alpha-synuclein (aSyn) in inclusions known as Lewy bodies (LBs) and Lewy neurites (LNs). Although Lewy pathology is prominently localised within brain stem, limbic, or cortical regions and is consistent with disease symptomology, inconsistencies between aggregate burden, cell type-specific vulnerability and disease severity are well documented [4, 16, 28, 45, 58, 61]. Therefore, whether Lewy pathology is the sole underlying factor for neuronal dysfunction and death in LBDs is unclear. Nevertheless, genetic studies linking mutations in the aSyn gene (SNCA) with PD [55] and an array of cellular and animal model studies support a causative role of aSyn in LBD pathology [14, 38].

aSyn is primarily a presynaptic protein, although initial studies had indicated that the protein may also be present within the nucleus [37], this has long been controversial and overlooked [26]. Indeed reports of nuclear aSyn (aSyn^Nuc^) have been undermined by the discovery that several commercial aSyn antibodies possess cross-reactivity with putative non-aSyn nuclear antigens [26]. However, increased support for the nuclear localisation of aSyn has been gained by studies utilising novel antibodies validated in aSyn knockout mouse tissue, fluorophore-coupled fusion proteins, and the use of nuclear isolation protocols [52, 54, 63, 71]. Although there is a lack of evidence supporting physiological aSyn^Nuc^ in humans, intranuclear aSyn inclusions are a pathological feature of multiple system atrophy (MSA) and thus supports the detrimental potential of altered aSyn^Nuc^ composition in human neurodegenerative diseases [34].

Studies using in vitro and in vivo models, suggests that aSyn^Nuc^ regulates a number of nuclear functions, interacting with DNA [52, 54, 59] and histones [20], consequently regulating gene transcription [13, 52, 54] and also DNA repair [59]. Critically, the overexpression of wild type or disease relevant aSyn mutants (e.g. A30P, A53T, or G51D) enhanced aSyn^Nuc^ levels, facilitate intranuclear inclusion formation, and induce impairments in gene transcription and nuclear import [9, 18, 29, 31, 62].

Although nuclear dysfunction may contribute to synucleinopathy pathology, the occurrence of aSyn^Nuc^ in human brain material is still controversial. Establishing the intra-nuclear presence of aSyn must be considered essential not only to progress our understanding of the molecular mechanisms associated with LBDs, but also to inform on the normal function of aSyn, both of which are instrumental for the design of future therapeutics.

## Materials and methods

### Tissue preparation

Post-mortem human brain tissue from clinico-pathologically confirmed cases of DLB (n = 22) and non-neurodegenerative diseases controls (n = 24) was obtained from the Newcastle Brain Tissue Resource. For histology, cingulate or lateral temporal cortex sections were prepared from the right hemisphere of the brain (4% paraformaldehyde immersion fixed, 6-weeks). Frozen temporal grey matter (Broadman area 21/22, middle and superior temporal gyrus, 500 mg) for subcellular fractionation, biochemical analysis, and MS was obtained from the contralateral left hemisphere of the brain, (snap frozen between copper plates at − 120 °C).

Disease conformation for each case was established by a review of clinical history following death and neuropathological assessment according to Lewy Body Braak staging [8], McKeith Criteria [42, 43] and the National institute of ageing—Alzheimer’s Association (NIA-AA) criteria [47], including neurofibrillary tangle Braak staging [7], Thal Aβ phases [65] and Consortium to Establish a Registry for Alzheimer’s Disease (CERAD) scoring [46] (Table 1 and Additional file 1: Table S1).

**Table 1:** Human tissue cohort

Disease	n	Sex (% male)	Age (years)	PMI (hrs)	NFT Braak stage	Thal Phase	CERAD	NIA-AA	LB Braak stage	McKeith Criteria
*Histology-qualitative*										
Con	6	66.70%	60–90	24–85	0-IV	0–3	neg-B	Low-inter	0	100%-No LB
			82 ± 4.5	55.3 ± 9.5	16.6%-0	16.6%-0	83.3%-neg	83.3%- low	100%-0	
					16.6%-I	16.6.%-1	16.6%-B	16.6%-inter		
					33.3%%- II	16.6%-2				
					16.6%-III	50%-3				
					16.6%-IV					
DLB	9	66.70%	72–91	10–99	I–III	0–4	*neg-B	Not-inter	*6	100%-Neo
			78.2 ± 1.9	55 ± 12.1	11.1%-I	14.3%-0	55.6%-neg	11.1%-not	100%-6	
					89.9%-III	14.3%-1	22.2%-A	55.6%-low		
						14.3%-2	22.2%-B	33.3%- inter		
						14.3%-3				
						42.9%-4				
*Histology – pS129 nuclear quantification*										
Con	12	58.30%	60–95	15–66	0-III	0–5	neg-A	Not-inter	0	100%-No LB
			81 ± 3.2	42 ± 5.4	25%-0	41.7%-0	91.7%-neg	41.7%-not	100%-0	
					50%-II	25%-1	8.3%-A	50%-low		
					25%-III	8.3%-2		8.3%-inter		
						8.3%-3				
						8.3%-4				
						8.3%-5				
DLB	12	91.60%	71–91	8–98	II–III	*0–5	neg-B	Not-inter	*4–6	16.7%-limbic
			78.5 ± 1.7	38.8 ± 8.8	41.7%-II	30%-0	60%-neg	20%-not	12.5%-4	83.3%-Neo
					58.3%-III	10%-1	10%-A	30%-low	87.5%-6	
						10%-2	30%-B	50%-inter		
						20%-3				
						10%-4				
						20%-5				
*Western blot*										
Con	9	55.60%	73–99	5–75	0-III	0–4	neg	Not-low	0	100%-No LB
			85.3 ± 2.7	31.7 ± 7.4	22.2%-0	22.2%-0	100%-neg	22.2%-not	100%-0	
					11.1%-I	22.2%-1		77.7%-low		
					55.6%-II	11.1%-2				
					11.1%-III	33.3%-3				
						11.1%-4				
DLB	8	75%	68–92	8–99	II–III	03-May	neg-B	Low-inter	05-Jun	100%-Neo
			78.5 ± 3	39.5 ± 11.9	37.5%-II	12.5%-3	62.5%-neg	87.5%-low	25%-5	
					62.5%-III	62.5%-4	25%-A	12.5%- inter	75%–6	
						25%-5	12.5%-B			
*Mass spectrometry*										
Con	3	33.30%	64–99	5–93	I–II	0–1	neg	Not-low	0	100%-No LB
			81 ± 10.1	41 ± 26	33.3%-I	66.7%-0	100%-neg	66.7%-low	100%-0	
					66.7%-II	33.3%-1		33.3%-inter		
DLB	3	100%	78–81	8–34	III	03-Apr	A-B	Low-inter	6	100%-Neo
			79.7 ± 0.9	22.7 ± 7.7	100%-III	33.3%-3	33.3%-A	33.3%-low	100%-6	
						67.7%-4	67.7%-B	66.7%-inter		

Human cases use for histology, western blot and mass spectrometry. Case are separated by disease classification according to non-diseased controls (Con) and dementia with Lewy bodies (DLB). Case numbers (n), sex, age, post-mortem interval (PMI), neurofibrillary tangle (NFT) Braak stage, Thal phase, Consortium to Establish a Registry for Alzheimer’s Disease (CERAD), the National Institute of Ageing—Alzheimer’s Association (NIA-AA) criteria, Lewy body (LB) Braak stage and McKeith criteria are provided. For age and PMI both range and mean ± SEM are provided. For numerical scores of pathology, range and percentage composition are given. For CERAD scores, negative (neg), A and B reported. For NIA-AA, not, low and intermediate (inter) risk for Alzheimer’s disease. For McKeith criteria, only percentage composition is given, where cases free of LBs (No LB) and neocortical predominate (Neo) are indicated. * = based on available data

Forebrain samples from three month-old aSyn knockout mice, generated as previously outlined [57], were snap frozen in liquid nitrogen, and stored at − 80 °C prior to use.

### Histochemical detection of nuclear aSyn

Paraffin embedded slide-mounted tissue sections (10 µm thick) were dewaxed (5 min, xylene), rehydrated (5 min, 99, 95, 75% ethanol) and washed in Tris-buffered saline (TBS; 5 mM Tris, 145 mM NaCl, pH 7.4). Optimal nuclear antigen detection was determined by subjecting sections to various antigen retrieval methods (Table 2). Sections were subsequently blocked in 5% normal goat serum (NGS) containing TBS (1 h, RT), labelled for phospho-Ser129 aSyn (mouse pS129 IgG2 [36], 1:1000 or EP1536Y [ab51253, Abcam], 1:500 dilution, with and without 5 × pS129-aSyn blocking peptide [ab188826]), pan aSyn (N-terminal directed, SYN303 [aa 2–12 Cat# 82,401, Biolegend], 1:500, non-amyloid β component directed Syn-1 [aa91-99, Cat# 610,787, BD Transduction Laboratories], 1:500), Histone H3 ([Cat# 3638S, Cell Signalling], 1:500) and/or NeuN ([ab104224, Abcam], 1:200) via an overnight incubation in primary antibody containing solution (5% NGS containing TBS, 4 °C) followed by secondary antibody incubation (1 h, RT, goat anti-rabbit alexa 594 and goat anti-mouse alexa 488 and or goat anti-mouse Ig2B 647, 1:500, Fisher Scientific). Autofluorescence was quenched (0.03% Sudan Black B in 70% ethanol, 5 min) and slides coverslipped with Prolong Diamond Mountant containing DAPI (Fisher Scientific). Sections were imaged via a wide-field fluorescence (Nikon Eclipse 90i microscope, DsQi1Mc camera and NIS elements software V 3.0, Nikon) or confocal (Lecia SP 8, LAS X software, Leica-microsystems) microscope.

**Table 2:** Antigen retrieval methods

Antigen retrieval	Method
Citrate	Submersion in pre-heated citrate buffer (10 mM Citric acid, 0.05% Tween 20, pH 6) with microwave heat-assisted antigen retrieval (800 W, 10 min)
Formic	Submersion in 90% Formic acid for 1 h at room temperature (RT)
Proteinase K	Slides incubated at 37 °C for 20 min in Proteinase K (0.6u/ml) containing TE buffer (50 mM Tris, 1 mM EDTA, 0.5% Triton X-100, pH 8)
EDTA + Formic	Submersion in pre-heated EDTA buffer (10 mM EDTA, pH 8) and subsequent heating in pressure cooker (heated under maximum pressure for 2 min) followed by submersion in 90% formic acid for 10 min at RT

Protocols for each of the evaluated antigen retrieval methods for nuclear antigen detection

### Analysis of pS129 nuclear immunoreactivity

Quantification of nuclear pS129, was performed using a Lecia SP8 confocal microscope and slides imaged via 63 × oil immersion lens (n = 12 DLB and 12 controls). For each case, 5 images from layers V-VI of the lateral temporal cortex were selected at random and sampled via Z-Stack imaging (0.3 µm step thickness). For analysis, Z-stacks of 21 images were summated using Z-project maximum intensity function of ImageJ (NIH), DAPI fluorescence was used to manually trace individual nuclei as regions of interest (ROI), which were grouped according to NeuN reactivity enabling the quantification of pS129 per ROI as per NeuN +ve and NeuN −ve nuclei. Mean values of fluorescence intensity, without threshold application, were calculated per image and then further averaged to provide a mean value of pS129 immunoreactivity for NeuN +ve and NeuN −ve cells per case, before being pooled according to disease (e.g. Control Cf. DLB). Comparisons between control and DLB cases were made via Mann–Whitney tests (GraphPad Prism, Ver 5) and correlative analysis between NeuN +ve and NeuN −ve within individuals performed via Spearman’s correlation (Prism), *p* < 0.05 was deemed significant. Additionally, frequency plots of pS129 immunoreactivity of all NeuN +ve and NeuN −ve nuclei were generated for control and DLB cases as a visual representation of nuclear changes at a cellular population level, using Prism.

### Tissue homogenisation and fractionation

Purified nuclear extracts were generated as per previously reported method [40]. Tissue blocks (250 mg) and individual cerebral hemispheres from aSyn knockout mice were lysed 1:16 (W:V) in nuclear extraction buffer (0.32 mM sucrose, 5 mM CaCl_2,_ 3 mM Mg(Ac)_2_ 10 mM Tris–HCl, 0.1% NP-40, pH 8, containing cOmplete protease inhibitor cocktail and phostop tablets, 1/10 ml, Sigma) via dounce homogenisation to generate crude whole tissue lysates. Cytoplasmic fractions were obtained from the centrifugation (800 rcf × 40 min, 4 °C) of crude lysates (500 µl) and retention of the resulting supernatant. Nuclei were isolated from the remaining lysates via sucrose gradient centrifugation (1.8 M Sucrose, 3 mM Mg(Ac)_2_, 10 mM Tris–HCl, pH 8, 107,000 rcf × 2.5 h, 4 °C). Pelleted nuclei were washed twice in PBS and resuspended in 500 µl 0.01 M phosphate buffer saline. Fractions were directly applied to microscope slides for immunochemical staining (as above) or frozen at − 80 °C.

### Immunoblotting analyses

Nuclear fractions, pre-treated with DNAse (50u/ml, 15 min RT, RQ1 DNAse, Promega) and cytoplasmic fractions, were adjusted to 80 ng/µl with LDS sample buffer (Fisher Scientific), reducing agent (Fisher Scientific) and dH_2_O and heated at 70 °C for 10 min. Samples (4 µg/lane for phospho-aSyn blots and 1.2 µg/lane for all other blots) were separated via SDS page electrophoresis (200 V, 35 min in MES buffer) and transferred to nitrocellulose membranes (0.2 µm) via Iblot (7 min, 20 mV; Fisher Scientific). Initial blots were optimised for aSyn retention by post-transfer heat treatment in 10 mM phosphate buffered saline (PBS; microwaved, 800 W, 5 min in boiling PBS) or via 30 min chemical fixation (4% paraformaldehyde, 30 min, RT). All subsequent aSyn blots were pre-treated via microwave-heat assisted protein retention (800 W, 5 min in boiling in 10 mM PBS), prior to being blocked in 5% milk powder containing Tris buffered saline with Tween-20 (TBST; 5 mM Tris, 145 mM NaCl, 0.01% Tween-20, pH 7.4, 1 h, RT). Membranes were stained for aSyn (pS129-aSyn, EP1536Y, 1:1000, Syn-1, 1:5000 and C-terminal directed MJRF1 [aa119-123, Cat# ab138501, Abcam], 1:5000) in TBST with 0.05% sodium azide overnight at 4 °C and labelled with secondary antibodies (goat anti-mouse HRP or goat anti-rabbit-HRP, 1:5000, in 5% milk powder containing TBST, 1 h, RT). Between incubation steps, blots were washed 3 × 5 min in TBST). Immunoreactivity was visualised via enhanced chemiluminescence (1.25 mM Luminol, 30 μM coumaric acid and 0.015% H_2_O_2_), images captured with a Fuji LAS 4000 with imaging software (Fuji LAS Image, Raytek, Sheffield, UK). Protein loading and fractionation purity was established via reprobing membranes with the cytoplasmic marker GAPDH (1:5,000, 14C10, Cell signalling) and the nuclear marker Histone H3 (1:5000). Western blots were quantified via area under the curve of immunoreactive bands (AUC; ImageJ) adjusted to the AUC of corresponding loading controls. Samples were processed in batches, each blot contained ≥ 3 control and DLB cases, allowing normalisation of measurements to control values within blot prior to pooling between blots. Comparisons were made via Mann–Whitney tests, *p* < 0.05 was deemed significant.

### Mass spectrometry analyses of fractionated tissue

#### Protein digestion

Frozen samples (250 mg) were fractionated as above, nuclear pellets were resuspended in 50 µl of 5% SDS containing PBS and cytoplasmic fractions were mixed 1:1 with 10% SDS solution to give a final 5% SDS concentration. Proteins were digested using S-Trap (Protifi) and samples cleared of DNA/RNA by heating to 95 °C and sonication. Samples were then reduced with TCEP (5 mM final concentration, 15 min incubation at 55 °C) and alkylated with Iodoacetamide (10 mM final concentration, 10 min incubation at RT). Resulting samples were acidified with 12% Phosphoric acid (final concentration of 2.5%, v/v), followed by addition of 6 vol. of loading buffer (90% methanol, 100 mM TEAB, pH 8) and loaded onto S-Trap cartridges. Cartridges were spun at 4000×g for 30 s and washed with 90% loading buffer × 3 and flow-through discarded. Retained proteins were trypsin digested (10:1 protein: trypsin), in 50 mM TEAB, pH8.5 for 3 h at 47 °C.

Peptides were eluted, first, with 50 µl of 50 mM TEAB, followed by 50 µl of 0.2% formic acid and finally with 50 µl of 50% acetonitrile and 0.2% formic acid.

### Detecting aSyn in subcellular fractions

To maximise the sensitivity/detectability of aSyn in the sub cellular fractions, a peptide level fractionation approach was used. A pooled sample (combined for controls and DLB cases) for each subcellular fraction was created by taking the volume for 20 µg of peptide solution from each sample, adding 20 µg of each sample to the same microcentrifuge tube. Peptides were dried by centrifugal evaporation. The near dried peptide pellet was dissolved in 50 µl, 20 mM ammonium formate, pH 8.0, sonicated for 5 min in a sonicating water bath then the solution was clarified by centrifugation at 13,000×g for 10 min at room temperature. The supernatant was transferred to a liquid chromatography (LC) vial with 200 µl insert. Alkaline reverse phase LC was carried out on a Thermo Ultimate 3000 UHPLC, with a 25 cm Phenomenex C18 column, 5 µM particle size, 100 Å pore size. The buffers used were A: ammonium formate, pH 8 and B: 100% (v/v) acetonitrile. 50 µl of peptide solution was injected and separated over a 40 min linear gradient from 2% buffer B to 40% buffer B at a flow rate of 300 µl/min. Fractions were collected every 1 min, with fraction 1 and 13, 2 and 14, 3 and 15 etc. collected into the same vial. This created 12 fractions. These samples were dried in a centrifugal evaporator to remove acetonitrile and ammonium formate. The dried peptides were dissolved in 15 µl of 3% Acetonitrile, 0.5% TFA, sonicated for 5 min in a sonicating water bath then the solution was clarified by centrifugation at 13,000 xg for 10 min at room temperature then transferred to an LCMS vial. All 12 fractions were acquired with the same LCMS parameters as below with ~ 1 µg of peptides loaded on column per fraction.

### Quantitative proteomics

For the determination of the abundance of aSyn and subcellular compartment purity controls, samples from controls and DLB were processed separately (giving 6 nuclear and 6 cytoplasmic subcellular fractions, from the 3 controls and 3 DLB cases) as per the s-trap method, as described in the previous section. On elution of peptides from the S-trap cartridge, samples were frozen and dried with centrifugal evaporator until at a volume of ~ 1 µl. The sample was dissolved in 0.1% Formic acid at a concentration of 1 µg/µl.

Peptide samples were loaded with 1 µg per LCMS run, peptides separated with a 125 min nonlinear gradient (3–40% B, 0.1% formic acid (Line A) and 80% acetonitrile, 0.1% formic acid (Line B)) using an UltiMate 3000 RSLCnano HPLC. Samples were loaded onto a 300 μm × 5 mm C18 PepMap C18 trap cartridge in 0.1% formic acid at 10 µl/min for 3 min and further separated on a 75μmx50cm C18 column (Thermo EasySpray -C18 2 µm) with integrated emitter at 250 nl/min. The eluent was directed into a Thermo Orbitrap Exploris 480 mass spectrometer through the EasySpray source at a temperature of 320 °C, spray voltage 1900 V. The total LCMS run time was 150 min. Orbitrap full scan resolution was 60,000, RF lens 50%, Normalised ACG Target 300%. Precursors for MSMS were selected via a top 20 method. MIPS set to peptide, Intensity threshold 5.0 e3, charge state 2–7 and dynamic exclusion after 1 times for 35 s 10 ppm mass tolerance. ddMS2 scans were performed at 15,000 resolution, HCD collision energy 27%, first mass 110 m/z, ACG Target Standard.

### Proteomic analysis

The acquired data was searched against the human protein sequence database, available from (https://www.uniprot.org/uniprot/?query=proteome:UP000005640), concatenated to the Common Repository for Adventitious Proteins v.2012.01.01 (cRAP, ftp://ftp.thegpm.org/fasta/cRAP), using MaxQuant v1.6.43. Fractions were assigned as appropriate. Parameters used: cysteine alkylation: iodoacetamide, digestion enzyme: trypsin, Parent Mass Error of 5 ppm, fragment mass error of 10 ppm. The confidence cut-off representative to FDR < 0.01 was applied to the search result file. Data processing was performed in Perseus [66]. All the common contaminants, reversed database hits and proteins quantified with less than 2 unique peptides were removed from the dataset. For quantitative proteomics, per subcellular fraction per case, intensity values for proteins were transformed to log2 and technical replicates averaged. Median was then subtracted within each sample to account for unequal loading and the width of the distribution adjusted. The dataset was filtered, keeping proteins with > 2 valid values in at least one experimental group. Remaining missing values were imputed from the left tail of the normal distribution (2StDev away from the mean, ± 0.3 StDev). These compositional values were used for principle component analysis of the two fractions and were used to demonstrate relative abundance of selected proteins known to be enriched with various cellular compartments as per The Universal Protein Resource Knowledgebase (UniProtKB [11]).

## Results

### In situ*** detection of aSyn***^***Nuc***^

**Fig. 1:**
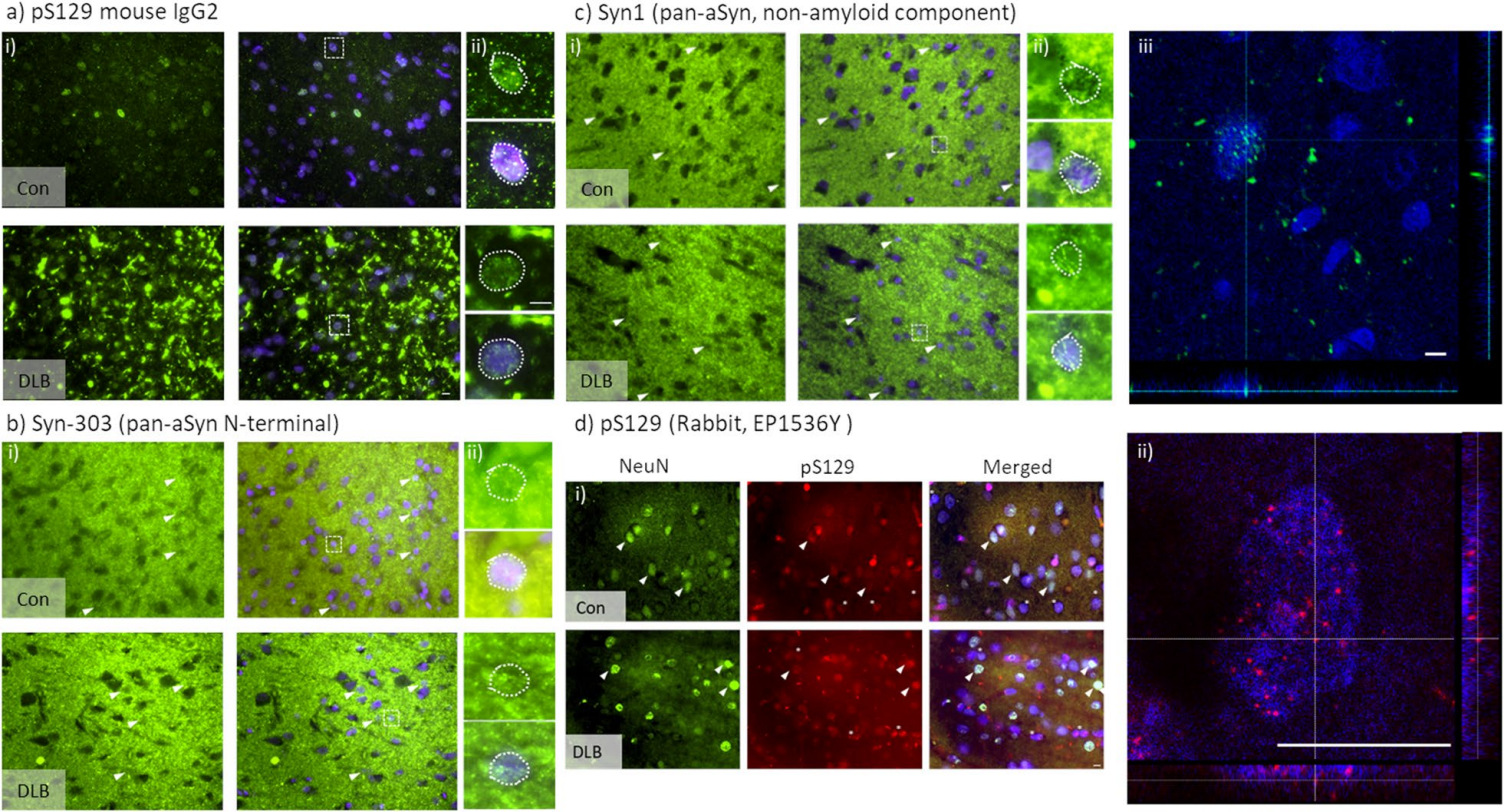
In-situ immunohistochemical detection of nuclear aSyn. Example micrograph images from the cingulate cortex of control (con) and dementia with Lewy body (DLB) cases of phospho-serine 129 positive aSyn (pS129) mouse IgG2 **(a)**, pan-aSyn N-terminal directed Syn-303 **(b)**, pan-aSyn non-amyloid component directed Syn1 **(c)** and NeuN and pS129 rabbit EP1536Y **(d)** immunoreactivity. All sections were pre-treated with EDTA + formic acid antigen retrieval, nuclei in sections were counterstained with DAPI. Images captured via 40× (**a–c, i**), 60 × objective lens (**d, i**) with wide-field fluorescence microscope and 20 × objective lens with 6 × digital zoom (**c, iii**) or 63× objective lens with 3 × digital zoom (**d, ii**) with confocal microscope. Expanded area inserts (**a–c, ii**, dotted line boxes in **i** are shown for aSyn immunoreactive alone and in combination with DAPI nuclear stain, where the nuclear outline is highlighted (dotted outline). Note frequent detection of intranuclear aSyn immunoreactivity, examples highlighted by arrow heads, in **d** NeuN-negative nuclei positive for aSyn are further denotes with an asterisk. Scale bar in i = 10 µm and ii and iii = 5 µm

A variety of antigen retrieval methods (Table 2) were initially evaluated for optimal nuclear antigen detection in cortical sections, using Histone H3 as a general nuclear marker. In comparison to individual treatment with citrate buffer, formic acid, or without pre-treatment, an antigen retrieval protocol combining pressurised heating in EDTA and 10 min submersion in 90% formic acid was found to yield the most robust H3 nuclear labelling (Additional file 4: Fig. S1). Consistent with the improved nuclear antigen access, the nuclear labelling of pS129 with mouse anti-pS129 was greatly enhanced following EDTA + formic acid pre-treatment, with only modest labelling of the nuclear compartment evident under the other treatments (Fig. 1a, Additional file 4: Fig. S2). In-situ aSyn^Nuc^ was further confirmed via the labelling of additional sections with N-terminal (Syn303) and the non-amyloid component (Syn-1) directed antibodies (Fig. 1b, c). Despite the prominent staining of the neuropil via pan-aSyn antibodies, numerous examples of positive aSyn immunoreactivity within nuclei were evident in all cases examined and orthogonal projections demonstrate multiple aSyn positive puncta within DAPI positive nuclei. Furthermore, staining with a second pS129 antibody (EP1536Y) together with the neuronal marker NeuN demonstrated intranuclear puncta evident in both NeuN positive neurons and NeuN negative non-neuronal cells of controls and DLB cases (Fig. 1d). Again, orthogonal confocal image projections confirmed ps129 aSyn positive puncta within DAPI positive nuclear structures (Fig. 1d). To confirm the specificity of EP1536Y staining for aSyn^Nuc^, similar incubations of human temporal cortex sections were performed in the presence of a pS129 aSyn blocking peptide. Such treatment abolished any notable immunoreactivity above that the no primary antibody control (Additional file 4: Fig. S3).

**Fig. 2:**
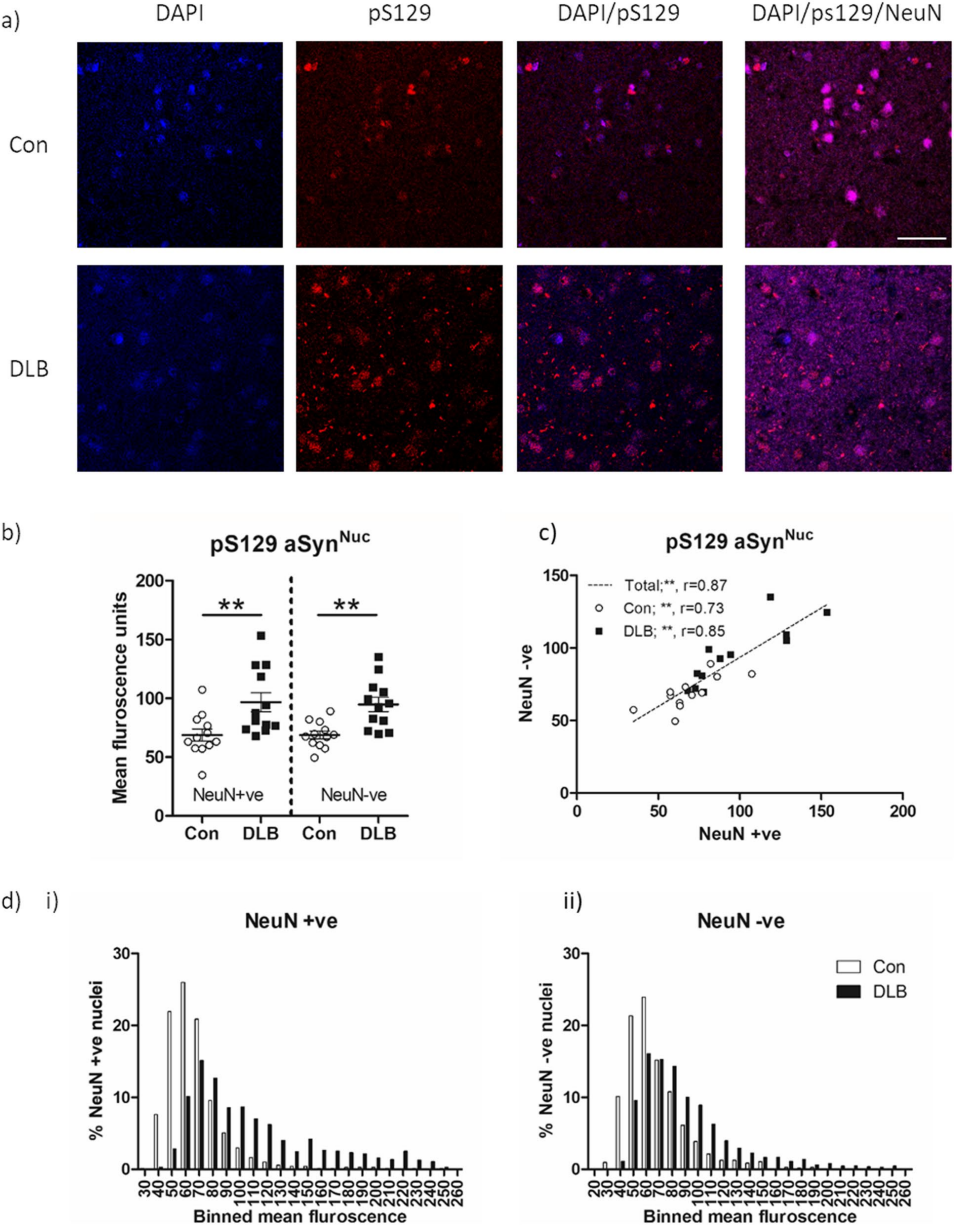
Quantification of nuclear phospho-S129 aSyn in temporal cortex of DLB and Control cases. Example of 60 × objective lens confocal images of lateral temporal cortex stained with phospho-serine 129 aSyn (pS129; EP1536Y) and NeuN antibodies with a DAPI nuclear co-stain **(a)**. Images from a control (Con) and dementia with Lewy body (DLB) case are shown. Note prominent pS129 nuclear stain in both cases, with additional cytoplasmic pathology in the form of Lewy neurites in the DLB case. Quantification of nuclear pS129 immunoreactivity in control and DLB cases as segregated according to NeuN reactivity is shown **(b)** alongside correlative analysis of nuclear pS129 levels between NeuN positive (NeuN +ve) and negative (NeuN −ve) nuclei **(c)**, with spearman’s correlation (r), given when analysed as a combined total single cohort or when divided into Con and DLB. Frequency plots of individual NeuN +ve (i) and NeuN −ve (ii) nuclei are additionally shown **(d)**, reporting the % nuclei of con and DLB cases within a of 20 arbitrary units. Data in c) reported as scatter plots of mean fluorescence arbitrary units per case with group mean ± SEM indicated and in **d)** also as mean fluorescence units, dotted line demonstrates best-fit line when all cases (con + DLB) are considered combined. ** = *p* < 0.01. Scale bar in a = 50 µm

Disease dependent alterations in aSyn^Nuc^ phosphorylation were quantified in a series of cases imaged via confocal Z-stack, and comparisons made between DLB and controls (Fig. 2a, Additional file 4: Fig. S4). In DLB tissue a significant elevation of pS129 immunoreactivity was observed in both NeuN +ve and NeuN−ve cells of layers V-VI of the lateral temporal cortex (Fig. 2b, *p* < 0.01, for both). Within cases, levels of pS129aSyn^Nuc^ correlated between NeuN +ve and NeuN −ve populations, both when considered across the entire cohort (Fig. 2c; r = 0.87, *p* < 0.01) but also when split into Con (r = 0.73, *p* < 0.01) and DLB (r = 0.85, *p* < 0.01) groups (Fig. 2c). Frequency analysis of the cellular populations further demonstrated a prominent rightward shift in pS129aSyn^Nuc^ immunoreactivity for both NeuN +ve and NeuN −ve cells (Fig. 2d i + ii) in DLB cases. Such an increase in pS129aSyn^Nuc^ profile suggests alterations occur throughout the cellular population as opposed to within a subpopulation of cells. No significant correlations were observed between mean pS129 levels with age or post-mortem delay, for either NeuN +ve or NeuN −ve values, when considered within the study cohort overall or when spilt according to controls or DLB groups (Additional file 3: Table S3).

### Detection of aSyn within isolated nuclei

**Fig. 3:**
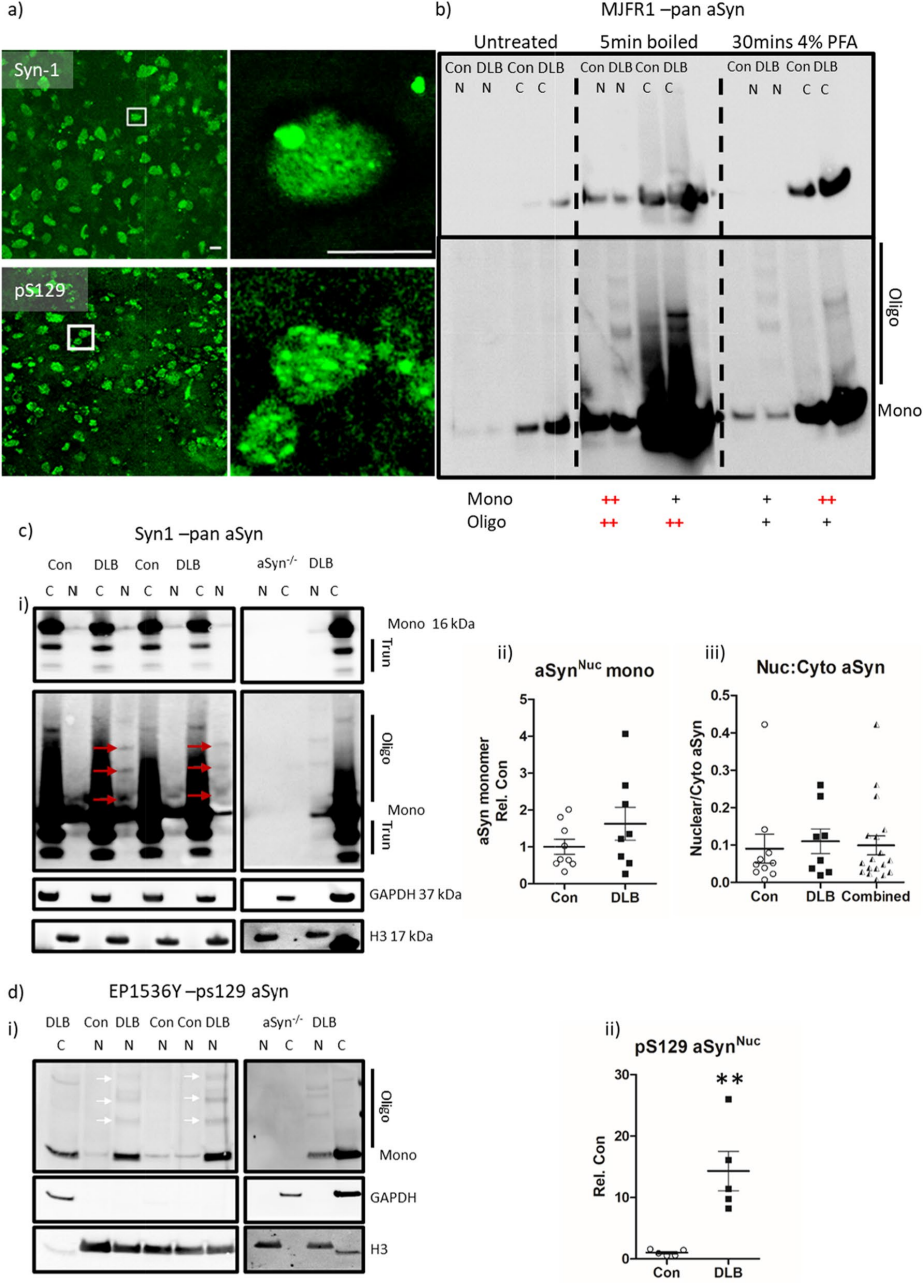
Quantification of nuclear aSyn pathology in cases of dementia with lewy bodies in isolated nuclei. **a** Representative confocal micrograph images of nuclei isolated from lateral temporal cortex (× 40 objective, inserts ii + iv taken with 10 × digital zoom, scale = 5 µm) stained for pan-aSyn (Syn-1, i + ii) and phospho-serine 129 aSyn (pS129, iii + iv). **b** Optimisation of immunoblot detection of aSyn from cytoplasmic (c) and nuclear (n) fractionates generated from control (Con) and dementia with Lewy bodies (DLB) cases. Comparisons of post-transfer membrane phosphate buffered saline heating (5 min boiling) or 4% paraformaldehyde chemical (30 min 4% paraformaldehyde) fixation with non-fixed (untreated) controls is shown, captured under optimal and overexposed settings. Monomeric (mono) and oligomeric (oligo) immunoreactivity is highlighted alongside a ranking of fixation methods-based retention of the aSyn species (+ = enhanced and++ = very enhanced). Quantification of **c** pan-aSyn via Syn-1, and **d** pS129 via EP1536Y, **(i)** example immunoblots following boiling fixation are shown at optimised and overexposed capture settings and mono, oligo and truncated (trunc) species are highlighted **(i)**. Loading controls of GAPDH and Histone H3 demonstrate fractionation purity and resulting immunoreactivity from similarly fractionated aSyn knockout mouse brain tissue (aSyn^−/−^) demonstrated antibody specificity. Quantification of nuclear aSyn monomers between DLB and controls cases is also shown **(ii)**, demonstrating increased pS129 Syn in DLB cases compared to controls (n = 5 per group), despite no change in total aSyn levels (n = 9 and 8 for con and DLB respectively) and no difference in the nuclear: cytoplasmic ratio of total aSyn **(c iii)**. Data shown as scatter plots, expressed relative to mean control values (Rel. Con) with mean ± SEM also indicated, ** = *p* < 0.01

Nuclear and cytoplasmic fractionations prepared from frozen temporal cortex were examined via western blot for aSyn. Pan and pS129-aSyn immunostaining of isolated nuclear preparations again confirmed the presence of aSyn^Nuc^ (Fig. 3a). Due to the low protein yield of the nuclear preparations, the membrane retention of aSyn was first optimised [32]. Post-transfer membrane boiling in PBS and chemical fixation with 4% paraformaldehyde enhanced aSyn immunoreactivity in both nuclear and cytoplasmic tissue fractions and revealed high molecular weight aSyn species present only in the nuclear fraction of DLB cases (Fig. 3b). Notably the two fixation methods favoured the retention of different aSyn species, such that paraformaldehyde treatment was optimal for cytoplasmic aSyn monomers, whilst PBS treatment was optimal for nuclear monomers and higher molecular weight species (Fig. 3b), likely suggesting differing chemical composition between pools of aSyn.

aSyn^Nuc^ was further confirmed in a larger number of cases, following the boiling of membranes in PBS and aSyn detection via pan-Syn1 antibody (Fig. 3c). In each sample the purity of the nuclear and cytoplasmic fractions was determined via Histone H3 and GAPDH controls (Fig. 3c). The levels of monomeric aSyn^Nuc^ were not significantly different between control and DLBs (Fig. 3d, *p* > 0.05), but were ~ tenfold lower than those in the cytoplasmic fractions, independent of disease status (Fig. 3c, 0.09 ± 0.04 cf. 0.11 ± 0.03 in controls cf. DLB respectively, *p* > 0.05). Longer exposures of Syn-1 blots again revealed the occurrence of higher molecular weight aSyn species of ~ 28 kDa, 42–45 kDa and 54–56 kDa consistent with the presence of nuclear aSyn oligomers in DLB cases (Fig. 3c, arrows). Similar results were also obtained using MJRF1 pan-aSyn antibody (Additional file 4: Fig. S5). The comparable levels of aSyn^Nuc^ monomer in nuclear fractions derived from control and DLB cases, argues against the detection of aSyn^Nuc^ as a consequence of the erroneous enrichment of aggregated aSyn due to fractionation. Both monomeric and oligomeric aSyn^Nuc^ were pS129 positive and the levels of pS129 reactive monomers were elevated in DLB cases compared to controls (14.3 ± 3.2-fold increase, *p* < 0.01, Fig. 2d). Critically, no immunoreactivity towards Syn-1, MJFR1 or pS129 was detected in similarly processed tissue from aSyn knockout mice (Fig. 3 and Additional file 4: Fig. S5). To further rule out the potential of cytoplasmic contamination of the nuclear fraction, analysis of GAPDH expression across cytoplasmic and nuclear fraction was conducted. Of the 13 cases investigated, quantifiable GAPDH signals within the nuclear fraction were detected in only 6 samples (~ 46%), at ~ 300 fold lower than that of cytoplasmic GAPDH (Additional file 4: Fig. S6), thus the magnitude of cytoplasmic contamination cannot account for the presence of aSyn within the nuclear fraction.

### Label-free detection of aSyn within isolated nuclear preparations

**Fig. 4:**
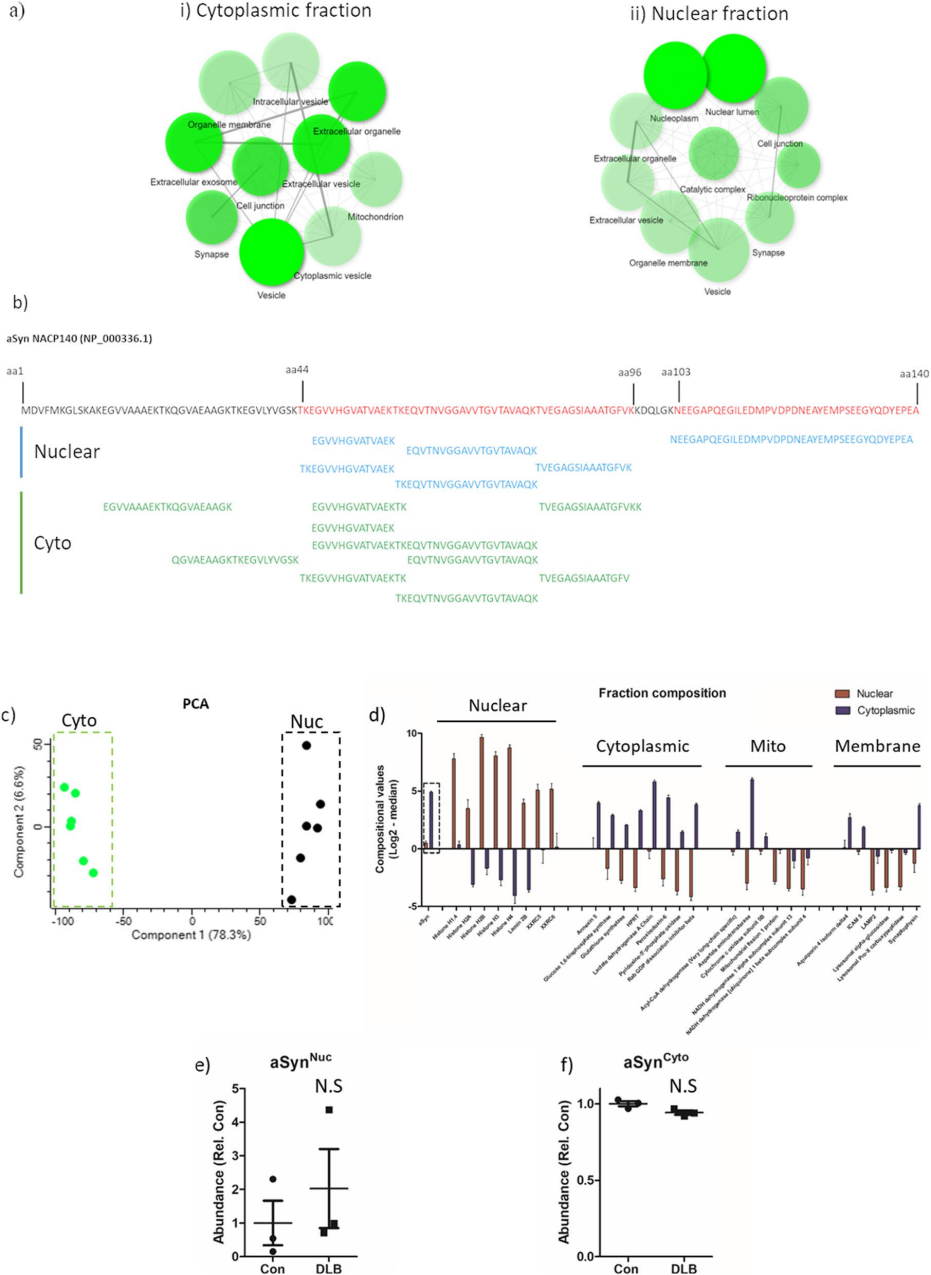
Mass spectrometry fractional analysis and aSyn peptide sequence alignment. **a** Gene ontology cell component enrichment analysis of mass-spectrometry identified proteosome from nuclear (**i**) and cytoplasmic fraction (**ii**) isolated from the lateral temporal cortex. Cell component networks are shown as produced from Shiny GO (V 0.66). For each node, darker shading represents significantly greater gene enrichment, node size indicates the number of genes aligning with the specific cellular component node and the thickness of connecting lines demonstrates the magnitude of overlapping genes between nodes. **b** Peptide mapping to the aSyn amino acid (aa) sequence, unique peptides recovered from mass spectrometry of nuclear fraction are shown in blue and those recovered from the cytoplasmic fraction shown in green. Full length aSyn sequence (NP_000336.1) is shown above with the sequence covered by peptides present in nuclear fraction shown in red. **c** Principal component analysis (PCA) of the relative abundance of proteins across pooled protein library, demonstrated clear distinction between cytoplasmic (Cyto) and Nuclear (Nuc) fractions. **d** Fractional composition of nuclear and cytoplasmic preparations, abundance of selective nuclear, cytoplasmic, mitochondrial (mito) and membrane proteins are show, alongside aSyn for comparison. Abundance values are also shown from nuclear (e; aSyn^Nuc^) and cytoplasmic (f; aSyn^Cyto^). Data expressed as compositional values (log^2^-median) + SEM. N.S = not significant

To address the limitations of antibody-based detection, both cytoplasmic and nuclear fractions were further investigated via MS. Subcellular fractions were pooled between cases (both control and DLB cases, initially) to generate a combined cytoplasmic and a second combined nuclear sample and peptide identification was performed in 12 step LC-fractionation enabled the generation of nuclear (~ 7500 proteins) and cytoplasmic (~ 5700 proteins) proteomic libraries. Gene ontology analysis (ShinyGO) of the resulting protein libraries reported significant enrichment of nuclear and cytoplasmic components with the respective nuclear and cytoplasmic preparations (Fig. 4a and Additional file 2: Table S2). With respect to aSyn, 7 peptides were detected in the nuclear subcellular fractionate, 6 of which were unique peptides specific to aSyn (NP_000336.1), these peptides are inclusive of two large sequences of the aSyn protein: aa44-aa96, which includes the aggregation prone non-amyloid β component region, and aa103-aa140, encompassing the disorganised C-terminal (Fig. 4b and Additional file 4: Fig. S7). For comparison 15 peptides, 12 of which were unique, were reported for the cytoplasmic fraction (Fig. 4b). To further validate the degree of purity within the nuclear fraction, quantitative proteomics was performed, to enable relative abundance measurements of all proteins detected in both nuclear and cytoplasmic fractions. Analysis of compositional abundance confirmed distinct proteomic profiles between the two fractions, cytoplasmic samples being clearly segregated from nuclear samples via principal component analysis (Fig. 4c). Comparison of abundance values for various subcellular specific proteins within the nuclear fraction demonstrated a marked enrichment of nuclear proteins and a diminution of proteins associated with the cytoplasm, mitochondria, and membranes (Fig. 4d). Comparatively compositional abundance scores relating to aSyn, although reduced in the nuclear fraction compared to the cytoplasmic fraction, scores were not of a similar magnitude of disparity (0.09 nuclear: cytoplasmic ratio) than those of cytoplasmic, mitochondria and membrane markers. Critically, of those proteins which demonstrated comparable abundance levels to aSyn within the cytoplasmic fraction, nuclear fraction values were much lower than that of aSyn ( cytoplasmic/nuclear abundance; aSyn 4.9 ± 0.08/0.47 ± 0.2; cytoplasmic lactate dehydrogenase A chain 5.8 ± 0.14/− 0.22 ± 0.6; mitochondrial aspartate aminotransferase 6 ± 0.13/− 2.98 ± 0.59 and pre-synaptic membrane synaptophysin 3.72 ± 0.18/− 1.275 ± 0.77) further arguing against the detection of aSyn within nuclear fractions as a consequence of cytoplasmic, or indeed mitochondria or membrane contamination. Comparisons between DLB and controls cases reported an increase in aSyn^Nuc^ in DLB cases (~ twofold higher in DLB compared to controls), although a high degree of variability was observed and the increase was not significant (Fig. 4e). Similarly, there was no difference in cytoplasmic aSyn abundance between DLB and controls (Fig. 4f).

## Discussion

Using a combined approach of immunohistochemistry, biochemistry and MS of autoptic human brain tissue, we have demonstrated the occurrence of physiological aSyn^Nuc^ in human brain cells. Furthermore, our study highlights a nuclear-centric alteration of aSyn which occurs alongside the cytoplasmic formation of LBs in DLB.

### Presence of aSyn in the nucleus

Although we are the first to systematically characterise aSyn^Nuc^ in human tissue, aSyn^Nuc^ has been reported at endogenous levels in brain tissue from mice, primates and originally in electric rays [20, 29, 37, 41, 49, 59, 71]. Likewise, aSyn^Nuc^ is evident in transgenic aSyn overexpression animals [39] and cell models [21, 44, 54, 63, 72], some studies of which have implemented reporter fusion proteins to negate potential non-specific antibody cross-reactivity [21, 44]. Combined the evidence supports a conserved physiological occurrence of aSyn^Nuc^ in the brain. Nevertheless, given that several aSyn antibodies demonstrate non-aSyn specific cross-reactivity [26], and that many neuropathological reports have failed to observe aSyn^Nuc^ in human tissue [4, 8, 33, 43], this issue has remained controversial. Here, we demonstrate the specificity of the EP1536Y pS129 antibody in human brain tissue, as per the abolition of staining in the presence of a blocking peptide and that the specific antigen retrieval method employed upon fixed tissue prior to immunohistochemical protocols is a key factor in aSyn^Nuc^ detection. As antigen retrieval methods typically employed for the visualisation of LB pathology, namely formic acid and proteinase K [5], were suboptimal for aSyn^Nuc^, the occurrence of aSyn^Nuc^ in the human brain may have been largely overlooked. Although, we have previously identified subtle intranuclear aSyn aggregates in DLB temporal cortex with conformational dependent antibodies KM51 and 5G4 staining as following an EDTA + formic acid pre-treatment [54] in line with a modified protocol as per BrainNet Europe guidelines [1]. Accordingly, aSyn^Nuc^ may be detectable with modest adaptation of existing diagnostic protocols. In relation to the biochemical detection of aSyn^Nuc^, the low levels of endogenous aSyn^Nuc^ in human brain tissue, may have previously precluded immunoblot quantification of aSyn^Nuc^. The loss of low molecular weight proteins, including aSyn, from transfer membranes can lead to target proteins falling below detectable concentrations without post-transfer fixation [32]. Accordingly, we evaluated a paraformaldehyde fixation method, previously evaluated for increased aSyn retention [32] as well as boiling membranes in PBS, a method more commonly used for β-amyloid detection [30]. As optimal conditions for the membrane fixation for any protein are likely dependent on its chemical composition [69], it should be noted that heating in PBS was more favourable for optimising aSyn^Nuc^ whilst paraformaldehyde was more favourable for monomeric cytoplasmic aSyn, suggesting a difference in chemical composition between cytoplasmic and nuclear pools of aSyn. Independent from antigen binding and retention, aSyn was detected in nuclear enriched fractions of human brain tissue, in all cases investigated, by label free MS. Despite the nuclear preparation enviably containing non-nuclear contamination, comparison of prominent cytoplasmic, mitochondrial and membrane proteins, clearly demonstrated a much-reduced presence within the nuclear fraction compared to the abundance of aSyn within this nuclear fraction. Thus, aSyn detection within nuclear fractions is unlikely to be a consequence of erroneous non-nuclear compartmental enrichment. Indeed, our data not only argues against a cytoplasmic source of aSyn, but also specifically against pre-synaptic contamination, where aSyn is most prominently enriched [6] and also mitochondria [53], where aSyn has also been reported to be present.

Whilst each method used in this study have limitations, such as antibody specificity, addressed via blocking peptides as well as the absence of immunoreactivity within aSyn KO mouse tissue and the potential for fractional contamination, addressed by comparative analysis of equally abundant non-nuclear proteins, the consistent detection of aSyn^Nuc ^ across methods collectively negates overall concerns of artificial aSyn^Nuc^ detection. Furthermore, the visual and fractional sub-cellular localisation of aSyn immunoreactivity, with both pan and phosphorylation specific antibodies, alongside, the apparent disease dependent alteration in aSyn^Nuc^ phosphorylation and oligomerisation, strongly supports its genuine occurrence.

### ***Physiological localization of aSyn***^***Nuc***^

aSyn positive nuclei were present in both immunohistochemistry and immunoblot experiments, in all cases examined, independent of disease status. Moreover, pS129 aSyn^Nuc^ was detected in both NeuN positive neurons and NeuN negative glial lineage cells, consistent with the low aSyn expression in astrocytes and oligodendrocytes [23, 48, 56, 64]. Such ubiquity of aSyn^Nuc^ implies its involvement in physiological processes. Accordingly, aSyn^Nuc^ is associated with genomic integrity, participating in DNA double strand break repair [59], and/or enhancing transcription of genes downstream from retinoic acid [13]. Critically, however, when overexpressed, aSyn^Nuc^ downregulates gene transcription as a consequence of inhibited histone acetylation [29, 52], and/or via direct interactions with gene promotors [62]. Genes associated with DNA repair [52, 54] and mitochondrial regulation [62], are among the affected transcripts which highlights the potential for dysfunctional aSyn^Nuc^ to engage in established neurodegenerative pathways.

### ***Disease-dependent modification of aSyn***^***Nuc***^

The disease dependent alteration of aSyn^Nuc^, comprising of hyperphosphorylated pS129 aSyn and higher molecular weight oligomeric aSyn species was evident in DLB cases. Phosphorylation [2, 19] and oligomerisation [22] of aSyn are widely associated with LB pathology and, therefore, the nuclear alteration is consistent with speculated pathological mechanisms [51]. However, comparable molecular weight banding of aSyn was not detected in the cytoplasm of matched cases of DLBs, suggesting the changes in aSyn^Nuc^ composition are unlikely to reflect changes in whole cell aSyn composition and instead represent novel aSyn conformers. Methodologically, it remains possible that the presence of aSyn oligomers within the nuclear fraction, may in part be due to the sedimentation of large insoluble aggregates from the brain tissue of DLB cases. However, such oligomers are typically observed within denaturing western blot preparations in the protein denaturing anionic SDS detergent or urea fractions which are known to solubilise larger aggregates [3]. Here, tissue lysis and nuclear isolation, was performed in the absence of SDS or Urea, employing only the non-protein denaturing non-anionic detergent NP-40 [27] at a very low concentration (0.1%), thus such solubilisation of larger aggregates seems unlikely. In any case, the potential for larger aggregate contamination of the nuclear fraction, does not detract from the robust detection of monomeric aSyn^Nuc^ in both controls and DLBs and nor the serine 129 phosphorylation of such monomers.

Both control and DLB cases demonstrated pS129 aSyn^Nuc^, albeit this was ~ 14-fold higher in DLB cases in western blots and apparent in controls when quantified via immunohistochemistry, although this was elevated in DLB cases. Thus, aSyn^Nuc^ phosphorylation, at least at low levels, is not overtly pathological. Indeed, cellular studies imply pS129aSyn^Nuc^ can be regulated endogenously by Polo-like 2 kinase activity [17] and promotes its nuclear import [54, 60]. aSyn^Nuc^ phosphorylation may further facilitate the protein’s recruitment to DNA damage, modulate transcriptional effects and protect against cellular stress [54, 59], whilst also preventing intranuclear oligomer formation [31].

Nevertheless, an overabundance of phosphorylated aSyn, may impede the proteins interaction with DNA, with potential consequences for DNA stabilization and repair [15]. In addition to the facilitation of nuclear import, further studies point to the increased nuclear retention of pS129 aSyn [21], suggesting that an elevation such phosphorylated species, without regulation may lead to excessive intranuclear aSyn accumulation. Indeed, it is possible that physiological phosphorylation of aSyn may be required for its nuclear export. In disease conditions where phosphorylated aSyn is excessively high, such regulatory mechanisms may be overwhelmed and thus subcellular intranuclear accumulation beyond a physiological range may in turn be detrimental to nuclear homeostatic processes. The determinantal consequences of altered nuclear aSyn content is particularly evident from *in-vivo* studies, where enhanced aSyn^Nuc^ levels results in the loss of dopaminergic neurons and motor impairments [29, 35] and by the toxicity associated with the nuclear translocation of aSyn in response to oxidative stressors [20, 62, 68, 73]. It is interesting to note that the increased pS129 aSyn^Nuc^ as per immunohistochemical quantification is seen not in a selective subpopulation of cells but is clearly seen in a rightward (increasing) shift in distribution of pS129 aSyn^Nuc^ levels of control tissue. Such alteration in distribution suggests that changes in pS129 aSyn^Nuc^ are not limited to neurons containing LB pathology, but instead are evident in many cells of the cortex in DLB and thus potentially indicates a disruption of nuclear homeostasis throughout the cellular population. Nevertheless we did not find robust evidence of an overall accumulation of total aSyn and thus perhaps associated nuclear dysfunction may closer relate to the altered ratio between phosphorylated and non-phosphorylated aSyn^Nuc^.

As a consequence of altered post-translational modification, the risk of self-aggregation for aSyn^Nuc^ in disease conditions is likely altered/increased [38]. The observed pS129 positive oligomeric aSyn species would support this and is consistent with our previous observations of intranuclear reactivity towards oligomer/aggregation specific aSyn antibodies in DLB cases and the formation of aSyn^Nuc^ oligomers in cellular overexpression systems [54]. Certainly, the nuclear environment would appear prone to aggregation, as DNA [10, 12, 24] and histones [20] accelerate aSyn aggregation. Although we did not detect overt signs of mature fibril inclusions, neuronal and oligodendrocytic intranuclear inclusions of aSyn filaments are a pathological feature of MSA, alongside aSyn cytoplasmic aggregates [34]. As part of the MSA disease process elevated pS129 aSyn^Nuc^ is considered as an early event and is assumed to precede the formation of mature aggregates [25, 70]. In any case, the occurrence of neuronal nuclear inclusions in the brain stem nuclei of MSA cases correlates with surviving neuron numbers, suggestive of a protective role for mature inclusions [50], which is in line with cellular toxicity being largely independent of overt aSyn aggregates in cell models [68] and that instead, at least within the nucleus, toxicity may be driven by intermediate aSyn oligomers [67].

## Conclusion

This study has addressed an outstanding question in the field confirming the nuclear occurrence of aSyn using an array of histological and molecular methods in human *post-mortem* tissue. Furthermore, we have identified that aSyn^Nuc^ is altered in DLB and manifests increased levels of putatively pathogenic post-translation modifications, such as pS129. Intriguingly, aSyn^Nuc^ is enriched for oligomeric species distinct from those in the cytoplasm. Given the potential role of oligomers in disease-associated toxicity, such species may drive cellular impairment and neurodegeneration associated with DLB.

## Supplementary Information

**Additional file 1: Table S1.** Individual Human cases. Diagnosis (Diag), age (in years), sex, post mortem interval (PMI, in hrs) and neuropathological assessment scores for neurofibrillary tangle (NFT) Braak stage, Thal phase, Consortium to Establish a Registry for Alzheimer’s Disease (CERAD), the National Institute of Ageing – Alzheimer’s Association (NIA-AA) criteria, Lewy body (LB) Braak stage and McKeith criteria are provided. For McKeith criteria, absence of Lewy pathology (No LB), Limbic predominate and Neocortical (neoctrx) predominate are indicated. Additionally, the use of each case in western blots (WB), multi-channel fluorescence histochemistry (His) and/or mass spectrometry (MS) is also listed. N.A = not available.

**Additional file 2: Table S2.** Fraction enrichment. Top 30 cellular component categories identified from proteomic analysis of nuclear and cytoplasmic tissue fractions as per ShinyGo database. Assigned category name, number of proteins associated genes detected out of total list are provided as well as false discovery rate (FDR).

**Additional file 3: Table S3.** Correlative analysis of age and post-mortem delay with nuclear pS129 aSyn intensity. Table reports Spearman’s correlation (r) and associated significance of nuclear pS129 aSyn for NeuN +ve and NeuN −ve cell types when correlated with age or post-mortem delay. Data is presented for total cohort and when spilt into control and DLB cases. N.S = not significant, p > 0.05.

**Additional file 4: Fig. S1.** Nuclear antigen accessibility following antigen retrieval methods. Representative images captured via a 40 × objective lens from cingulate cortex from control cases stained for Histone H3 with DAPI nuclear stained. Comparison of staining with citrate, formic acid and EDTA + formic acid clearly demonstrates optimum nuclear labelling following a combined treatment of EDTA and formic acid. Scale bar = 10 µm. **Fig. S2.** Comparison of nuclear aSyn detection under different antigen retrieval method. Example micrograph images (× 40) from dementia with Lewy body (DLB) cases of a) phospho-serine 129 positive aSyn (pS129) mouse IgG2 immunoreactivity. Sections were pre-treated prior to antibody staining with Citrate buffer (a), Formic acid (b), Protienase K (c) and EDTA + Formic acid (d) methods of antigen retrieval. Expanded area inserts (ii, dotted line boxes in i) are shown for both pS129 alone and in combination with DAPI nuclear stain, where the nuclear outline is highlighted (dotted outline). Note robust detection of punctate intranuclear staining of pS129 following EDTA + Formic acid-based antigen retrieval. Scale bar in i = 10 µm and ii = 5 µm. **Fig. S3.** Specificity of EP1536Y pS129 antibody immunoreactivity. Demonstration of control temporal cortex sections, stained either with EP1526Y phospho-antibody, with or without blocking peptide or with no primary, following EDTA + formic acid antigen retrieval pre-treatment, all sections stained with secondary antibody and nuclei co-labelled with DAPI. Scale bar = 10 µm. **Fig. S4.** Nuclear phosphorylated s129aSyn in control and Dementia with lewy body cases. Micrographs captured via 20 × objective of temporal cortex tissue from control (Con) and Dementia with Lewy body (DLB) cases. Sections stained for pS129 aSyn (EP1536Y) and neurons (NeuN) with a DAPI nuclear stain. Scale bar = 50 µm. **Fig. S5.** Quantification of cytoplasmic proteins within nuclear fractions. Example western blots of GAPDH immunoreactivity of cytoplasmic (C) and nuclear (N) fractionates (a) without (i) and with (ii) enhanced contrast to allow for visualisation of GAPDH within the nuclear fraction. Comparative analysis between GAPDH immunoreactivity from cytoplasmic and those sample in which GAPDH was above detection threshold (~ 46%) indicated ~ a 300 fold dilution of cytoplasmic components in the nuclear fraction (b). **Fig. S6.** Detection of nuclear aSyn via pan-aSyn antibody MJRF1. Example western blots of pan-aSyn MJFR1 immunoreactivity of cytoplasmic (c) and nuclear (N) fractionates from control (con) and cases of dementia with Lewy bodies (DLB) (1.8 µg/lane). Monomeric aSyn is shown under optimised exposure conditions, with large panel depicting monomeric and oligomeric aSyn species captured following overexposure of the blot. Antibody specificity was confirmed by means of similar probing of tissue fractionates generated from aSyn knockout mice (aSyn−/−). Cytoplasmic and nuclear loading controls GAPDH and Histone H3 are also shown. Note faint appearance of high molecular weight aSyn species in the nuclear fraction only in DLB cases (arrows) only in the nuclear fraction. **Fig. S7.** Mass spectrometry detection of aSyn in the nuclear fraction. Annotated spectra for each of the recovered peptides, b and y ions are highlighted accordingly.

## Data Availability

All data sets are available from the corresponding author upon request.

## Abbreviations

aSyn: Alpha-synuclein
aSyn^Nuc^: Nuclear alpha-synuclein
CERAD: Consortium to establish a registry for Alzheimer’s disease
DLB: Dementia with Lewy bodies
LBDs: Lewy body diseases
LBs: Lewy bodies
LC: Liquid chromatography
LNs: Lewy neurites
MS: Mass-spectrometry
MSA: Multiple system atrophy
NIA-AA: National institute of ageing-Alzheimer’s Association
PBS: Phosphate buffered saline
PD: Parkinson’s diseases
TBS: Tris buffered saline
TBST: Tris buffered saline with Tween
