# Supplementary material for: Nuclear alpha-synuclein is present in the human brain and is modified in dementia with Lewy bodies
Source: Acta Neuropathol Commun. 2022 Jul 6;10:98. doi: 10.1186/s40478-022-01403-x (PMC9258129; doi:10.1186/s40478-022-01403-x)
Supplement: Supplementary file 3 — Additional file 3: Table S3. Correlative analysis of age and post-mortem delay with nuclear pS129 aSyn intensity. Table reports Spearman’s correlation (r) and associated significance of nuclear pS129 aSyn for NeuN +ve and NeuN −ve cell types when correlated with age or post-mortem delay. Data is presented for total cohort and when spilt into control and DLB cases. N.S = not significant, p > 0.05. [file 40478_2022_1403_MOESM3_ESM.docx]

| Cell type | Age | PMD |
| --- | --- | --- |
| Total Cohort | | |
| NeuN +ve | r=0.72, N.S | r=-0.058, N.S |
| NeuN -ve | r=-0.034, N.S | r=0.005, N.S |
| Control cases | | |
| NeuN +ve | r=0.056, N.S | r=-0.011, N.S |
| NeuN -ve | r=-0.088, N.S | r=0.400, N.S |
| DLB cases | | |
| NeuN +ve | r=0.47, N.S | r=-0.077, N.S |
| NeuN -ve | r= 0.319, N.S | r=-0.203, N.S |

Supplemental figure 3- Correlation of pS129 aSyn immunoreactivity. Table reports Spearman’s correlation (r) and associated significance for NeuN +ve and NeuN -ve cell types when correlated with age or post-mortem delay. N.S = not significant, p>0.05.
