# Supplementary material for: Nuclear alpha-synuclein is present in the human brain and is modified in dementia with Lewy bodies
Source: Acta Neuropathol Commun. 2022 Jul 6;10:98. doi: 10.1186/s40478-022-01403-x (PMC9258129; doi:10.1186/s40478-022-01403-x)
Supplement: Supplementary file 4 — Additional file 4: Fig. S1. Nuclear antigen accessibility following antigen retrieval methods. Representative images captured via a 40 × objective lens from cingulate cortex from control cases stained for Histone H3 with DAPI nuclear stained. Comparison of staining with citrate, formic acid and EDTA + formic acid clearly demonstrates optimum nuclear labelling following a combined treatment of EDTA and formic acid. Scale bar = 10 µm. Fig. S2. Comparison of nuclear aSyn detection under different antigen retrieval method. Example micrograph images (× 40) from dementia with Lewy body (DLB) cases of a) phospho-serine 129 positive aSyn (pS129) mouse IgG2 immunoreactivity. Sections were pre-treated prior to antibody staining with Citrate buffer (a), Formic acid (b), Protienase K (c) and EDTA + Formic acid (d) methods of antigen retrieval. Expanded area inserts (ii, dotted line boxes in i) are shown for both pS129 alone and in combination with DAPI nuclear stain, where the nuclear outline is highlighted (dotted outline). Note robust detection of punctate intranuclear staining of pS129 following EDTA + Formic acid-based antigen retrieval. Scale bar in i = 10 µm and ii = 5 µm. Fig. S3. Specificity of EP1536Y pS129 antibody immunoreactivity. Demonstration of control temporal cortex sections, stained either with EP1526Y phospho-antibody, with or without blocking peptide or with no primary, following EDTA + formic acid antigen retrieval pre-treatment, all sections stained with secondary antibody and nuclei co-labelled with DAPI. Scale bar = 10 µm. Fig. S4. Nuclear phosphorylated s129aSyn in control and Dementia with lewy body cases. Micrographs captured via 20 × objective of temporal cortex tissue from control (Con) and Dementia with Lewy body (DLB) cases. Sections stained for pS129 aSyn (EP1536Y) and neurons (NeuN) with a DAPI nuclear stain. Scale bar = 50 µm. Fig. S5. Quantification of cytoplasmic proteins within nuclear fractions. Example western blots of GAPDH immunoreactivi [file 40478_2022_1403_MOESM4_ESM.pdf]

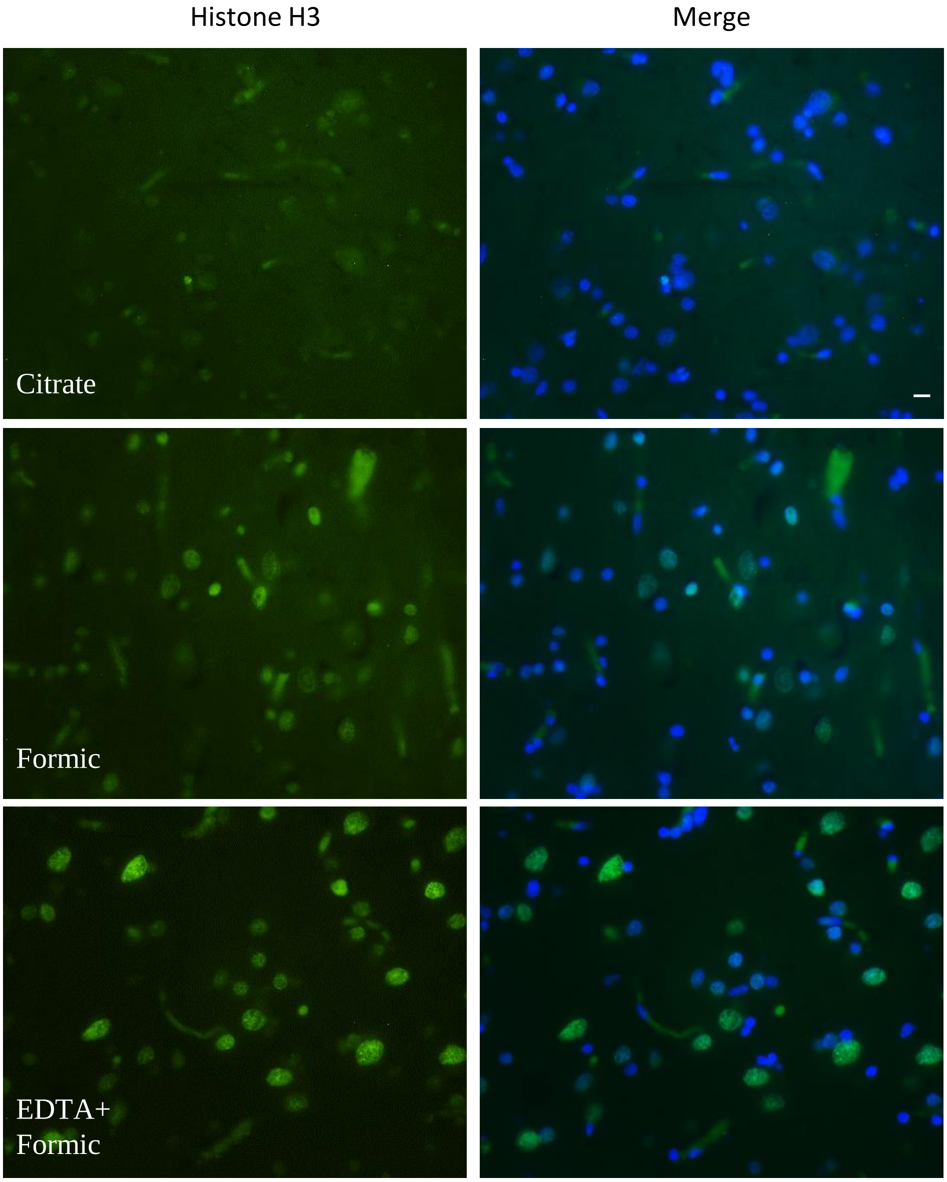

Supplemental Figure 1. Nuclear antigen accessibility following antigen retrieval methods. Representative images captured via a 40x objective lens from control cases stained for Histone H3 with DAPI nuclear stained. Comparison of staining with citrate, formic acid and EDTA + formic acid clearly demonstrates optimum nuclear labelling following a combined treatment of EDTA and formic acid. Scale bar = 10 µm.

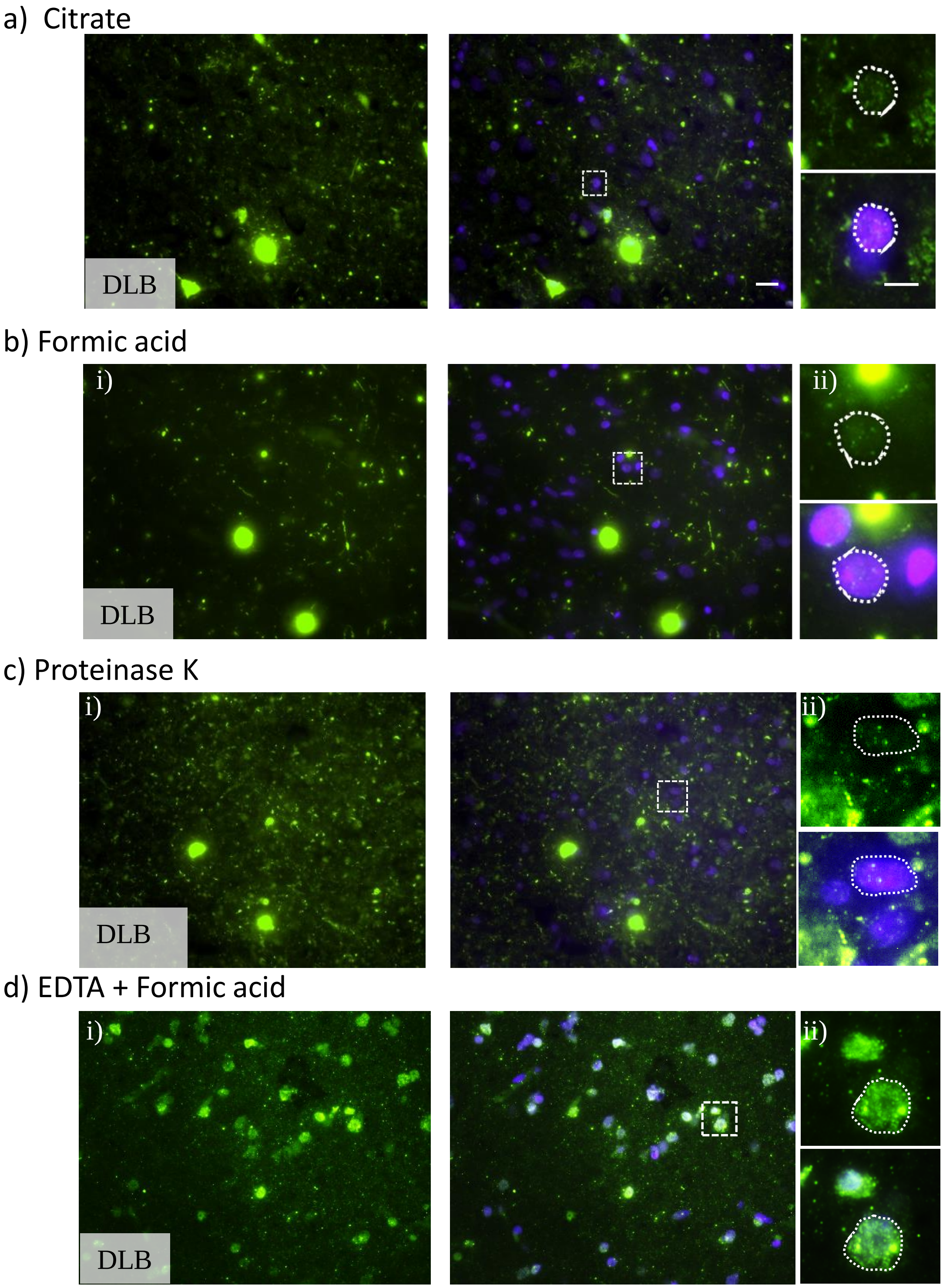

Supplemental Figure 2. Comparison of nuclear aSyn detection under different antigen retrieval method. Example micrograph images (x40) from control (con) and dementia with Lewy body (DLB) cases of phospho-serine 129 positive aSyn (pS129) mouse IgG2 immunoreactivity. Sections were pre-treated prior to antibody staining with Citrate buffer (**a**), Formic acid (**b**), Protienase K (**c**) and EDTA + Formic acid (**d**) methods of antigen retrieval. Expanded area inserts (ii, dotted line boxes in i) are shown for both pS129 alone and in combination with DAPI nuclear stain, where the nuclear outline is highlighted (dotted outline). Note robust detection of punctate intranuclear staining of pS129 following EDTA + Formic acid-based antigen retrieval. Scale bar in i = 10 µm and ii =5 µm.

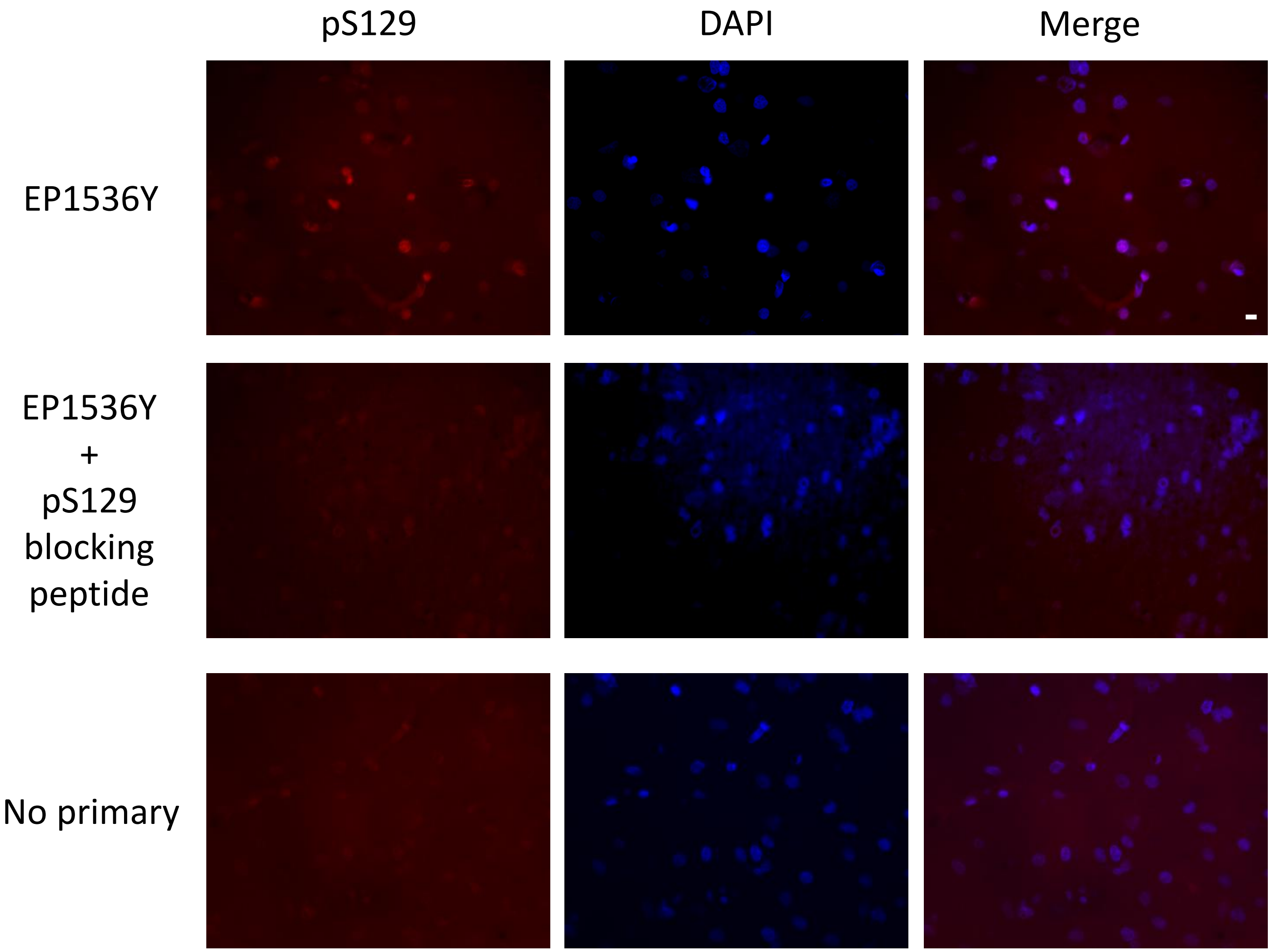

Supplemental Figure 3. Specificity of EP1536Y pS129 antibody immunoreactivity. Demonstration of control temporal cortex sections, stained either with EP1526Y phospho-antibody, with or without blocking peptide or with no primary, following EDTA + formic acid antigen retrieval pre-treatment, all sections stained with secondary antibody and nuclei co-labelled with DAPI. Scale bar =10 µm.

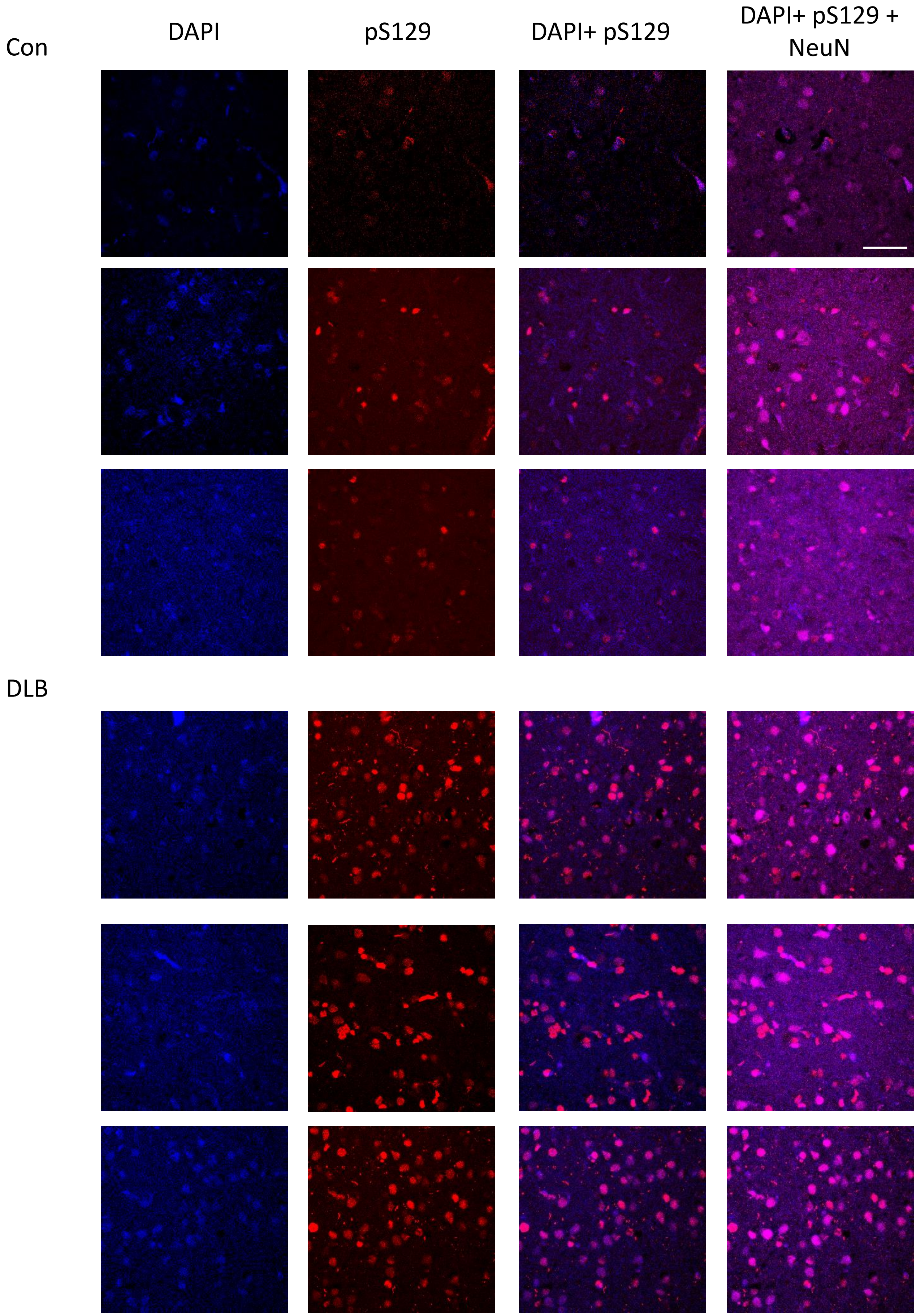

Supplemental Figure 4. Nuclear phosphorylated s129aSyn in control and Dementia with lewy body cases. Micrographs captured via 20x objective of temporal cortex tissue from control (Con) and Dementia with Lewy body (DLB) cases. Sections stained for pS129 aSyn (EP1536Y) and neurons (NeuN) with a DAPI nuclear stain. Scale bar = 50µm.

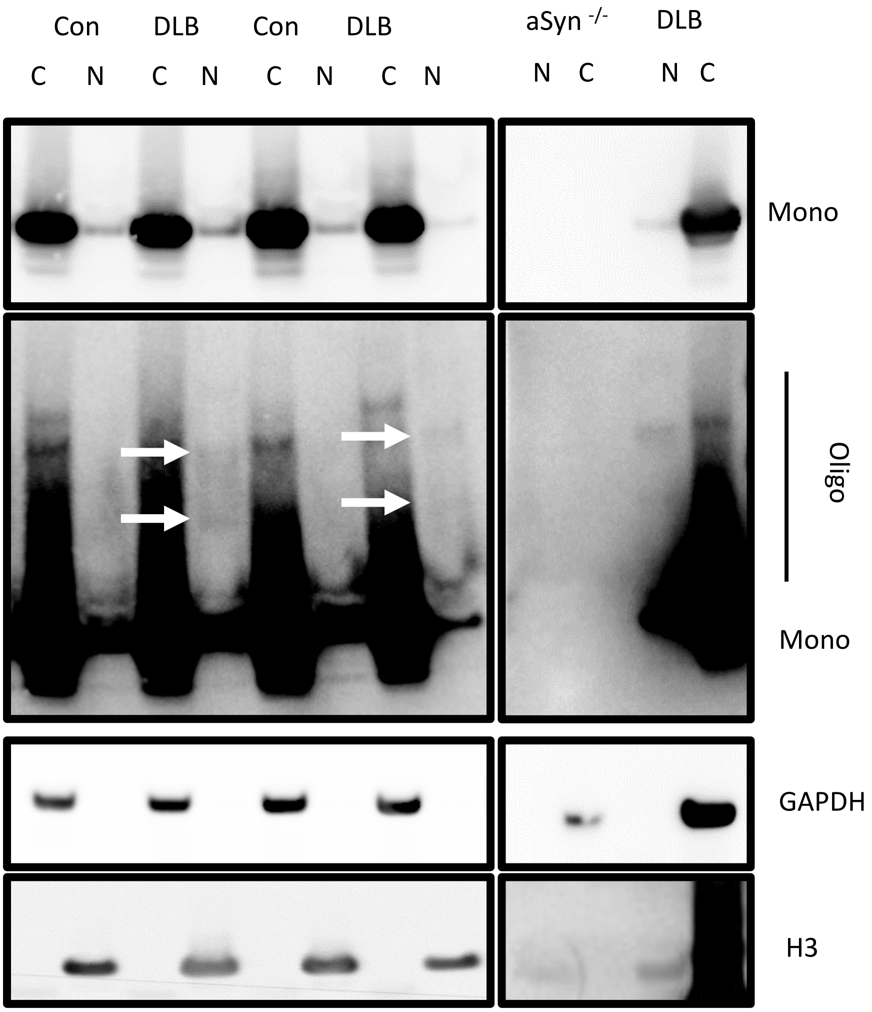

Supplemental figure 5 .Detection of nuclear aSyn via pan-aSyn antibody MJFR1. Example western blots of pan-aSyn MJFR1 immunoreactivity of cytoplasmic (c) and nuclear (N) fractionates from control (con) and Dementia with Lewy body (DLB) cases (1.8µg/lane). Monomeric aSyn is shown under optimised exposure conditions, with large panel depicting monomeric and oligomeric aSyn species captured following overexposure of the blot. Antibody specificity was confirmed by means of similar probing of tissue fractionates generated from aSyn knockout mice (aSyn$^{-/-}$). Cytoplasmic and nuclear loading controls GAPDH and Histone H3 are also shown. Note faint appearance of high molecular weight aSyn species in the nuclear fraction only in DLB cases (arrows) only in the nuclear fraction.

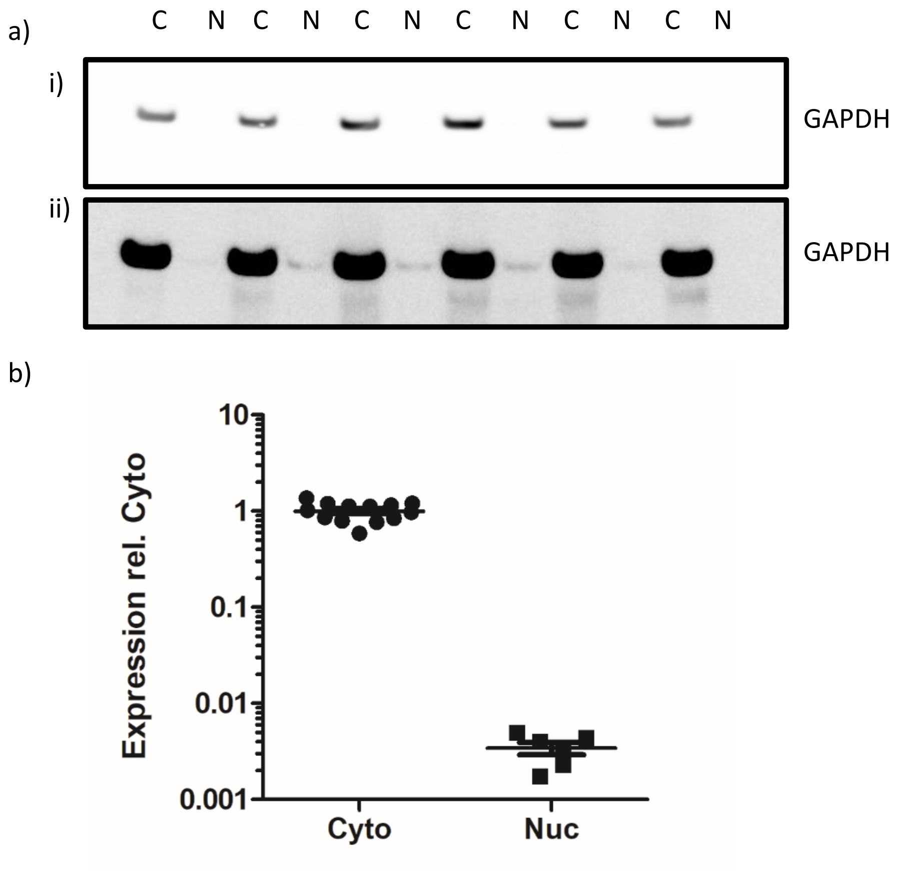

Supplemental figure 6. Quantification of cytoplasmic proteins within nuclear fractions. Example western blots of GAPDH immunoreactivity of cytoplasmic (c) and nuclear (N) fractionates (a) without (i) and with (ii) enhanced contrast to with enhanced contrast to allow for visualisation of GAPDH within the nuclear fraction (**a**). Comparative analysis between GAPDH immunoreactivity from cytoplasmic and those sample in which GAPDH was above detection threshold (~46%) indicated ~ a 300 fold dilution of cytoplasmic components in the nuclear fraction (**b**).

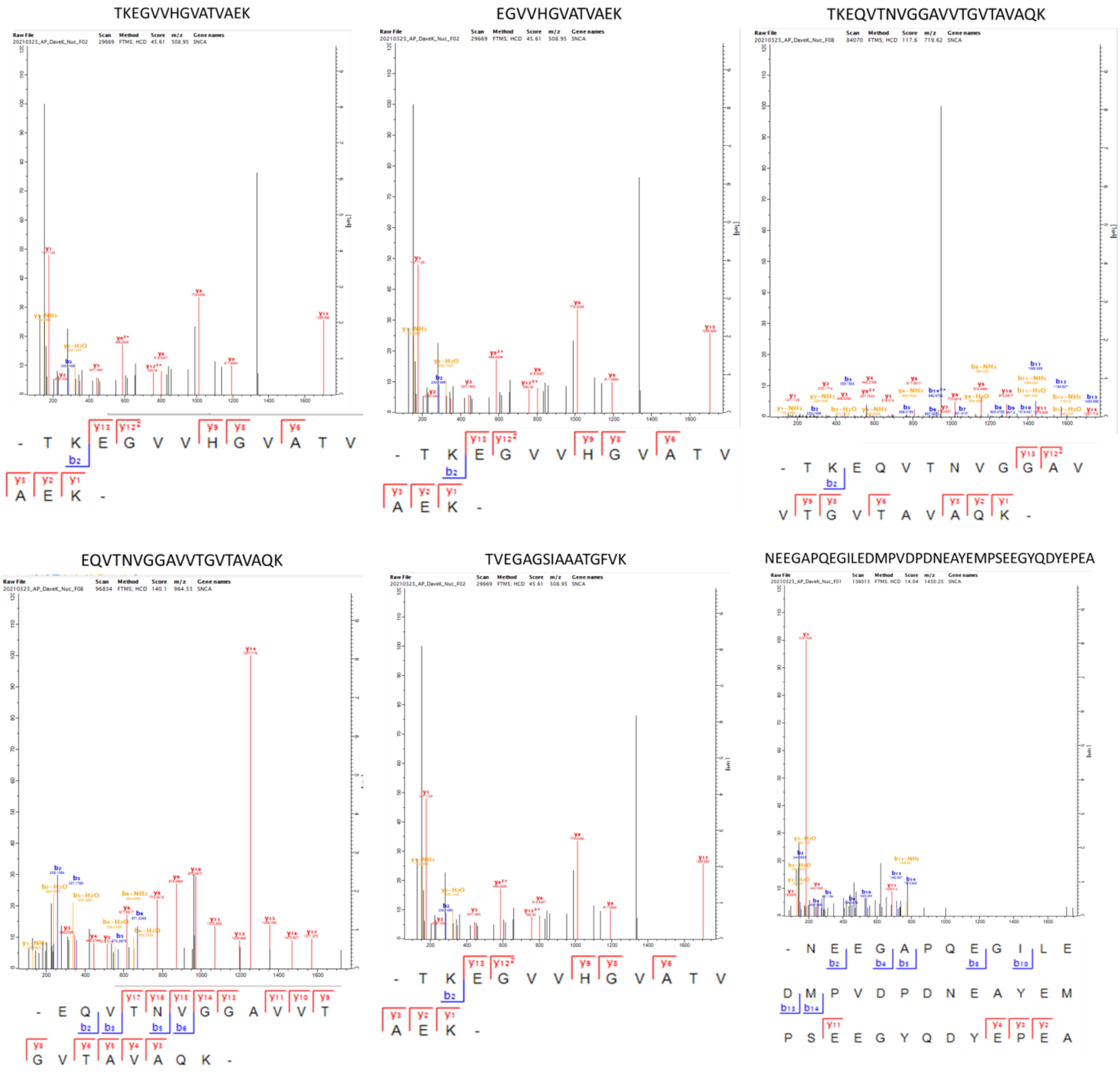

Supplemental figure 7. Mass spectrometry detection of alpha-synuclein in the nuclear fraction. Annotated spectra for each of the recovered peptides, b and y ions are highlighted accordingly.
